# Epidemiology of US patients with short bowel syndrome-associated intestinal failure: A claims database analysis

**DOI:** 10.1016/j.intf.2025.100325

**Published:** 2025-11-10

**Authors:** Ahan Ali, Gail Mitchell, Mark Gallivan, Jeff Henderson, Kishore Iyer

**Affiliations:** a Trinity Life Sciences, Waltham, MA, USA; b VectivBio, AG, Basel, Switzerland; c Mount Sinai Medical Center, New York, NY, USA

**Keywords:** Short bowel syndrome, Parenteral support, Real-world evidence, Claims database analysis, Epidemiology

## Abstract

**Background:**

After intestinal resection, patients with short bowel syndrome (SBS) have inadequate intestinal function, leading to malabsorption of nutrients and fluids, and in more extensive cases intestinal failure, requiring parenteral support (PS). Epidemiology analyses based on insurance claims have lacked diagnosis code specificity, leading to risks of misclassification during patient identification. The recent ICD-10 codes for SBS and intestinal failure (IF) could mitigate this. The objective of this claims-based study was to characterize the annual prevalence of patients with SBS dependent on PS, i.e. patients with SBS-associated IF (SBS-IF) from 2019 to 2021 in the USA.

**Methods:**

We conducted a retrospective claims data analysis to identify patients with SBS-IF using the Komodo Healthcare Map™ database. Inclusion criteria were at least two insurance claims for both chronic and continuous nutrition, six months apart, a malabsorption diagnosis, and either gastrointestinal surgery or congenital bowel disorder. Annual prevalence estimates were calculated using diagnosis rates from the cohort projected to the US population by age and gender.

**Results:**

In 2021, estimated US point prevalence of SBS-IF was ∼12,000 individuals, corresponding to approximately 36/million population (overall), with stable sex (2/3 female) and age distributions across years. From 2019 to 2021, prevalence increased by > 24 %. Sensitivity analyses using a broader definition produced an upper bound approaching 30,000 individuals (higher per-million rate accordingly).

**Conclusions:**

This claims-based algorithm requiring sustained PS plus clinical face-validity signals (malabsorption and relevant surgical/congenital history) yielded consistent, contemporary estimates of SBS-IF prevalence in the US, supporting downstream epidemiology and health-services analyses.

## Highlights

• SBS-IF epidemiology understanding was limited by lack of specific diagnosis codes.
• A claims data analysis identified patients with SBS-IF using recent ICD-10 codes.
• US prevalence was 12,000 patients in 2021.
• Prevalence increased by > 24 % from 2019 to 2021.
• A relaxed patient definition showed an up to 30,000 annual cases.

## Introduction

In adults, short bowel syndrome (SBS) is a rare, severe, and debilitating condition primary resulting from surgical resection of large portions of the small intestine typically caused by irreparable gastrointestinal (GI) damage due to inflammatory bowel disease, ischemia, neoplasms, or physical/abdominal trauma [1], [2]. Several definitions of SBS exist, based on length of remaining small intestine; the ESPEN guidelines mention less than 200 cm as the threshold [3], with reports suggesting that a residual length of 150 cm or less may be more appropriate [4].

The most common symptoms of SBS include diarrhea, abdominal pain, and weight loss, resulting in dehydration and malnutrition [1], [4], [5], [6].

Patients with more extensive resections or disease may require intravenous fluid or nutrition support, i.e. parenteral support (PS), meeting the definition for intestinal failure (IF) [7]. These patients have a reduction in gut function below the minimum necessary for the absorption of macronutrients, water and electrolytes; they cannot maintain protein-energy, fluid, electrolyte, or micronutrient balance when on a conventional normal diet [1], [8]. This leads to chronic dependence on PS, which consists of parenteral nutrition (PN) and/or intravenous (IV) hydration, to maintain health, growth, and survival [1], [4].

Although PS is essential to meet nutrient and fluid requirements, its chronic use is associated with serious, sometimes fatal complications, including catheter-related thrombosis, occlusion, and bloodstream infections, venous thrombosis, metabolic bone disease, renal dysfunction, bacterial overgrowth, and intestinal failure-associated liver disease [1], [9], [10], [11], [12], [13]. PS is also associated with a significant psychosocial, financial and technological treatment burden, impairing patients’ quality of life, as well as a significant burden on healthcare systems, resulting in indirect costs such as disability and lost work productivity for both patients and caregivers [1], [12], [14], [15].

SBS is associated with a high risk of mortality, ranging from 15 % to 47 % depending on patient’s age, and underlying disease [16].

The exact prevalence of SBS in the USA is unknown despite several efforts in the past decade due to under-reporting and a lack of reliable patient databases and diagnosis codes [4] as well as a lack of awareness of the condition in some geographical regions [17].

It has been estimated that 10,000–20,000 people in the USA have SBS-IF, i.e. a point prevalence of approximately 45 per million [18], although this figure may under-estimate the true prevalence of this condition. Indeed, based on other studies, the reported prevalence of SB-IF could range from 0.4 to 25.0 per million people across Europe and the USA [5], [11], [19], [20], [21]. A study of the prevalence, characteristics, and management of patients with chronic IF in the USA in 2012–2020 based on PS claims and healthcare utilization identified 24,048 patients, equivalent to 75 patients per million [22]. However, this analysis did not focus on SBS, therefore further analyses are need to better estimate the prevalence of SBS-IF.

Identifying the true prevalence of SBS-IF is crucial to characterize the clinical and economic burden of the disease and improve outcomes in these patients, but it is a challenge when employing administrative insurance claims, as diagnosis codes lack the specificity to reliably capture these patients. Previous retrospective analyses of large insurance claims databases have used a combination of International Classification of Diseases revision 10 (ICD-10) codes, rather than codes specific to SBS, to identify patients with SBS-IF [7]. This means that the current target account list for providers is incomplete, based on an inconsistent patient identification algorithm and gaps in the datasets used [11], [21], [23], [24]. However, it is important to note that new ICD-10 codes specific to SBS, SBS with intestinal failure, SBS with colon in continuity and SBS without colon in continuity have been approved and released in October 2023 and will hopefully improve diagnostic capture in the future [25].

The primary objective of this study was to improve the accuracy of the current estimates for the projected prevalence of patients with SBS-IF in the USA, by modifying the existing rules-based algorithm and employing stringent protocols for patient identification. A secondary objective of the study was to identify and characterize demographics and clinical characteristics of patients with SBS-IF.

## Methods

### Study design and data source

This real-world, retrospective cohort study was designed to estimate disease prevalence of patients with SBS-IF and characterize demographic and clinical characteristics of patients, including utilization of PS (PN and/or IV hydration). The study employed 2019–2021 data from the Komodo Healthcare Map™ claims database, capturing adjudicated medical and pharmacy insurance claims for approximately 100–120 million unique enrollees per year from over 70 health plans across the USA, and is representative of the US healthcare system for the *insured* US population, as it covers all types of payers (commercial, Medicare, Medicaid, and other payers) [26]. The Komodo database curates and links patient data from different sources to cover the entire patient journey (e.g., medical, pharmacy, claims, electronic health records, and laboratory results) on a continuous basis, using patient-matching algorithms to link all associated claims (both closed and open claims) at the patient level [26]. It has the ability to capture patients who switch plans, thus providing an enriched, longitudinal dataset with approximately 150 million patients meeting the ‘closed’ claims, for an overall patient volume of over 320 million patients over 3 years [26], [27]. To prevent double counting of patients, it maps each patient journey across different insurance programs and time [26]. The Komodo database has previously been validated in several therapy areas [28], [29].

### Analysis cohort definition

**Fig. 1:**
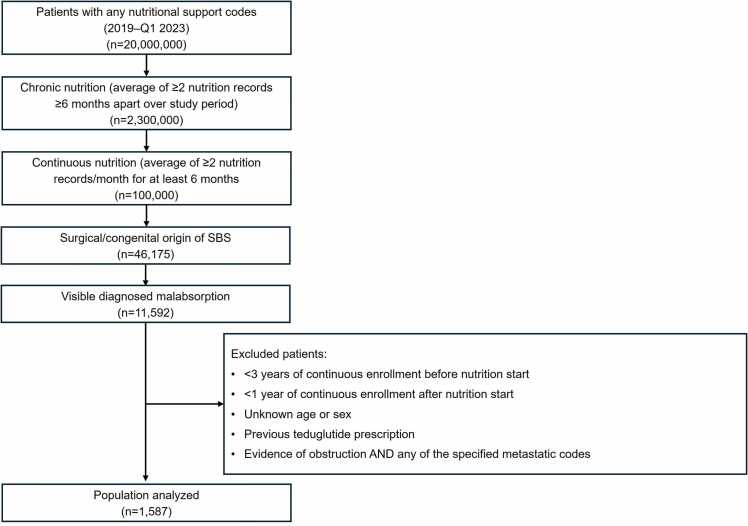
Patient disposition.

The primary cohort of patients with SBS dependent on PS was constructed using multi-factorial criteria to ensure a robust definition was applied to the database. Treating physicians were consulted to assess validity, along with ensuring clinical relevance of patient identification criteria, which was additionally supported through an assessment of the literature [12], [27], [30], [31]. Primary inclusion criteria for the study are outlined in Table 1 and Fig. 1 and detailed below:

• Nutrition requirement: at least one nutrition (PN or IV hydration) insurance claim (since January 1, 2016)
• Chronic nutrition criteria: at least two insurance claims for nutrition at least six months apart
• Continuous nutrition criteria: at least two insurance claims for nutrition per month (where the insurance claims were at least five days apart) in one-year intervals from 2019 to 2021
• Evidence of malabsorption: at least one insurance claim with a malabsorption diagnosis occurring before a patient’s first observed insurance claim for PN or IV hydration
• Evidence of SBS dependent on PS: either a history of any GI resection, post-surgical malabsorption or post-surgical complication diagnosis, or congenital abnormality of the intestine or necrosis. In addition, patients needed at least one diagnosis (e.g., post-surgical complication diagnosis) or surgery record occurring prior to initiation of nutrition support, and continuous enrollment in the year of patients’ index date (first observed PN or hydration claim date after surgery) and the subsequent two years (for a total of 3 years of continuous enrollment).

**Table 1:** Patient inclusion and exclusion criteria.

**Rules for patients with SBS dependent on PS**	**Details**	**Rationale for inclusion in the primary patient definition**	**Alternative definition 1** **(PN required, no malabsorption requirement)**	**Alternative definition 2** **(No malabsorption requirement)**
**Nutrition** requirement	Patient must have at least one **PN OR IV hydration**	Required to define patients with **intestinal failure**	Require insurance claims for PN only	Yes
**‘Chronic’** nutrition criteria	Patient must have at least 2 insurance claims for nutrition that are **at least 6 months** apart	Required to define patients as **chronic** intestinal failure (versus patients with an acute injury/nutrition requirement)	Yes	Yes
**‘Continuous’** nutrition criteria	Patient must average at least **2 insurance claims for nutrition per** month consecutively for at least 6 months	Based on refrigeration needs for nutrition products and at-home storage capabilities in the average home, patients with at-home nutrition **receive new nutrition supplies every 1–2 weeks** (i.e., at least twice a month)	Yes	Yes
Evidence of **malabsorption**	Patient must have at least one insurance claim with a **malabsorption diagnosis** in their visible claim history	A patient journey analysis of patients receiving teduglutide showed **that > 95 % of patients have diagnosed malabsorption** in their claim history	Not Required	Not Required
Evidence of **‘SBS dependent on PS’**	Patient must have evidence of one or more of the following: • History of any **gastrointestinal surgery** • Post-surgical malabsorption or **post-surgical complication** diagnosis • **Congenital** intestine malformation or necrosis diagnosis	Ensures patients with chronic intestinal failure identified (via the four rules identified above) have intestinal failure **resulting from surgical SBS**	Yes	Yes

IV, intravenous; PN, parenteral support; PS, parenteral support; SBS, short bowel syndrome.

Patients without full, closed continuous enrollment from 2018 to 2021 and without known age or gender were excluded; patients under 3 years of age were also excluded.

Sensitivity analyses were explored using alternative definitions for SBS dependent on PS to provide a range of plausible population sizes, with less stringent criteria to increase sample size. Alternative definition 1 did not require a malabsorption diagnosis and required at least one PN insurance claim instead of two. Alternative definition 2 also did not require a malabsorption diagnosis and required at least one insurance claim for PN or IV fluid.

### Statistical analysis

**Fig. 2:**
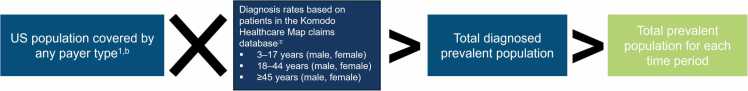
Projected prevalent population of patients with SBS-IF^a,^[29]^a^Process repeated for each defined time period. ^b^Commercial, Medicare, Medicaid, and other payers. ^c^Diagnosis rates calculated for each time period by age and gender grouping. PS, parenteral support; SBS, short bowel syndrome; US, United States.

Prevalence estimates were calculated in one-year intervals from 2019 to 2021. The numerator of the diagnosis rates was calculated for each calendar year across all payer channels by each age and gender grouping (Fig. 2). The denominator of the diagnosis rates was all patients in the Komodo Healthcare Map™ insurance claims database continuously enrolled for each calendar year and the previous three calendar years. Projections were performed to extrapolate diagnosis rates to the US population estimates by age and gender, applying the same diagnosis rate per year calculated from the Komodo Healthcare Map™ claims to the US population in each respective year reported by the US Census Bureau [32]. Projection estimates were computed for the age groupings of 3–17 years, 18–44 years, and ≥ 45 years.

Patient characteristics were assessed on a patient’s index date. Categorical patient characteristics were presented as counts and percentages; continuous variables were presented as means, standard deviations, medians, and interquartile. The dataset was extracted and analyzed in SQL (Snowflake Inc., San Mateo, CA, USA) and processed in R (version 4.1.3).

## Results

### Prevalence estimates of patients with SB-IF– primary cohort

A total of 44 million patients receiving nutritional support or with a GI disease-related diagnosis were identified from the Komodo Healthcare Map™ claims database between 2016 and 2022. During the study period (2019–2021), the annual prevalence of patients who met the primary inclusion criteria increased from 952 patients in 2019, to 1195 in 2020, and 1381 in 2021. When extrapolated to the US population, the prevalence was 9450, 10,824, and 11,803 patients for 2019, 2020, and 2021, respectively. This corresponds to a 24 % overall growth; year-on-year, this corresponds to 13 % in 2020 and 9 % in 2021. This ultimately yielded a prevalence rate of approximately 36 cases per million US inhabitants (9 cases per million for pediatric individuals and 26 cases per million for adults).

### Patient demographics – primary cohort

Based on the results for the year 2021, patients with SBS-IF were primarily White (48 %) and female (66 %), while patients aged 45 years or over constituted the largest age group (41 %), followed by patients in the 3–17 years (30 %) and patients in the 18–44 years (29 %) cohorts (Table 2). Similar trends for gender and age were observed in 2019 and 2020. Prevalence increased in all age groups between 2019 and 2021, with the greatest increase seen in the 3–17 years cohort (251 patients in 2019, increasing to 419 patients in 2021). Based on 2021 data, projections provide an estimated 3105 patients with SBS-IF aged 3–17 years and 8260 patients aged 18–65 years.

**Table 2:** Patient demographics and characteristics in 2021 – primary cohort.

	2019 Patients (n = 952)	2020 Patients (n = 1195)	2021 Patients (n = 1381)
Age (n, %)			
3–17	251 (26)	364 (30)	419 (30)
18–44	276 (29)	343 (29)	394 (29)
45–64	300 (32)	354 (30)	408 (30)
65–84	121 (13)	129 (11)	153 (11)
85 +	4 (0)	5 (0)	7 (1)
Gender (n, %)			
Female	621 (65)	797 (67)	911 (66)
Male	331 (35)	398 (33)	470 (34)
Nutrition Code Combination (n, %)^a^			
At least one EN claim	415 (44)	539 (45)	589 (43)
At least one IV hydration claim	689 (72)	833 (70)	997 (72)
Only PN claims	136 (14)	162 (14)	177 (13)
PN and IV hydration claims	231 (24)	298 (25)	342 (25)
PN and EN claims	127 (13)	200 (17)	207 (15)
PN, EN, and IV hydration claims	182 (19)	214 (18)	253 (18)

EN, enteral nutrition; IV, intravenous; PN, parenteral nutrition.

a Columns sum to > 100 % as patients can count under first two rows and last three rows.

Among female patients in 2021, 46 % were 45 years of age or older, 33 % were 18–44 years old, and 22 % were 3–17 years old. Male patients were younger, with the largest cohort being the 3–17 years cohort (47 %), followed by the 45 years old and over (32 %) and 18–44 years (20 %) cohorts.

### Comorbidities and prescribed medications – primary cohort

Based on data from 2021, patients with SBS-IF were diagnosed with a range of comorbidities, including GI disorders (83 %), abdominal pain (80 %), fluid and electrolyte disorders (72 %), and kidney/renal disease (24 %) (Table 3). Patients were also frequently diagnosed with infections, most commonly bacterial infections (41 %), urinary tract infections (30 %), and skin or subcutaneous tissue infections (24 %). Furthermore, 54 % of patients had a stoma. The most frequently prescribed medications were antiemetics (70 %), followed by antibiotics (50 %) and pain killers/opioids (45 %) (Figure S1).

**Table 3:** Comorbidities and infections using the Clinical Classifications Software Refined groupings of patients with sbs-if in 2021 – primary cohort.

	2021 Patients n= 1381
Comorbidities, %	
Gastrointestinal disorders	83
Abdominal pain	80
Fluid and electrolyte disorders	72
Nausea and vomiting	59
Esophageal disorders	58
Aplastic and other unspecific anemia	55
Respiratory Disease	56
Fever	51
Cardiovascular Disease	48
Musculoskeletal Pain	46
Kidney/Renal Disease	24
Infections, %	
Bacterial infection	41
Urinary tract infection	30
Skin/subcutaneous tissue infection	24
Respiratory infection	21
Viral infection	20
Fungal infection	17
Intestinal infection	13

Diagnosed using the Clinical Classifications Software Refined groupings [41]

PS, parenteral support; SBS, short bowel syndrome.

When assessing gender and age, adult males were less likely to experience nausea and vomiting than adult females, while these symptoms showed no differences in frequency among pediatric patients based on gender (Table S1). The rates of GI disorders generally decreased with age in both males and females; the observed increases in rates of fluid and electrolyte disorders suggest that these GI disorders were fluid and electrolyte disorders. Furthermore, adult females were co-medicated with ondansetron and antibiotics at higher rates than males, with the highest rates generally seen among females aged 18 years and over (Table S2). In pediatric patients, ondansetron and cephalexin were used at lower rates than in adults, whereas rates of amoxicillin were generally higher.

### Alternative cohorts

Two alternative definitions for identification of patients with SBS-IF were applied. Alternative definition 1 (which did not require a malabsorption diagnosis and required at least one PN insurance claim instead of two) led to a projected US prevalence of 15,317 patients, translating into 46 cases per million US inhabitants. Projections based on alternative definition 2 (which also did not require a malabsorption diagnosis and required at least one insurance claim for PN or IV fluid) resulted in a US prevalence of 29,886 patients, translating into 90 cases per million US inhabitants. Consequently, prevalence projections for alternative definitions 1 and 2 translated into a 30 % and 153 % increase, respectively, over the projected US estimates for the primary cohort in 2021.

When comparing the primary cohort with the alternative definitions, patient demographics were largely consistent; however, alternative definition 2 had fewer pediatric patients (23 %) compared with the primary cohort (30 %) and alternative definition 1 (29 %) (Table S3). Furthermore, a lower percentage of patients with GI disorders was observed using either alternative definition, while similar rates of abdominal pain, fluid and electrolyte disorders, nausea and vomiting, and esophageal disorders, were observed with the alternative definitions compared with the primary cohort.

## Discussion

Due to the lack of epidemiologic data for patients with SBS-IF, providing US disease prevalence, healthcare providers and decision-makers have not had the necessary awareness of the condition or data to inform patient access policies. Indeed, contemporary epidemiologic estimates may influence decision-making around coverage of therapies, aid in strategically planning for future healthcare needs, and inspire further research due to increased awareness of the economic burden of the condition.

Epidemiologic analyses of SBS-IF employing administrative insurance claims have been challenging, due to the lack of specific SBS ICD-10 codes to accurately identify patients in the US until October 2023 [25]. Consequently, stringent criteria were developed for this analysis, to identify patients with SBS-IF while mitigating the risk of false positives, considering multiple clinical factors, and employing input from treating physicians to ensure validity.

Due to the lack of coding precision, previous analyses have used a combination of diagnosis codes and evidence of a PS prescription to identify relevant patients with SBS-IF. Mundi et al. examined the characteristics of patients with chronic intestinal failure by capturing patients with at least two PS prescriptions within 6 months and a relevant diagnosis, resulting in an estimated prevalence of 75 patients with chronic intestinal failure per million US inhabitants over an 8-year study period [22]. By comparison, the current analysis applies a more stringent definition by identifying patients with SBS-IF and requiring at least two nutrition insurance claims per month for at least 6 months, which resulted in a narrower estimated prevalence of 11,803 or 36 patients with SBS-IF per million US inhabitants in 2021. These stringent criteria are likely to result in an under-estimate, as it is likely that some patients with SBS-IF get only one PN prescription per month and some may not even get one PN prescription per month. Indeed, a subset of patients with SBS receiving PN may be stable enough only to get PN prescriptions every 6 weeks. Additionally, for patients with multiple diagnoses (primary, secondary, tertiary), the coding physician may not have got to the malabsorption diagnosis.

Analysis of patient demographics from 2019 to 2021 revealed a greater proportion of female than male patients with SBS-IF (66 % vs 34 %), with higher diagnosis rates increasing with age groups. This may be due to the greater prevalence of Crohn’s disease in females [32]. Another explanation could be that female patients comprise 80 % of patients undergoing weight loss surgery, which can lead to SBS due to the extensive resection of the small bowel and post-operative complications [33]. Furthermore, age distribution varied by gender, with more male patients in the pediatric cohort. The slight predominance of male pediatric patients with SBS observed in our study (27 % vs 43 %) is supported by the literature. In a systematic review of pediatric patients with SBS, 67 % of patients were male and in a real-world study in Finnish pediatric patients with SBS, 63 % were male [34], [35]. This may be partly explained by the higher prevalence of rare congenital malformations such as omphalocele and Hirschsprung’s disease in male pediatric patients; however, additional research would be needed to further contextualize these findings [36], [37].

Based on results in the primary cohort in 2021, the most prescribed medications were antibiotics, likely due to the high rates of infections in this population, particularly in patients with stoma, who are at increased risk of infection [38].

Alternative, less stringent criteria for defining the cohort were explored to capture a broader patient cohort that more closely reflects historical epidemiological analyses, leading to prevalence of 15,317–29,886 patients, translating into 46 and 90 cases per million US inhabitants, respectively [5], [7], [11], [19], [20], [21]. This offers healthcare decision-makers a valuable upper-bound estimate pertaining to the *potential* US patient population who may be eligible for and benefit from treatment.

The current analysis showed modest year-over-year prevalence increases from 2019 to 2021. It is possible that more cases of SBS are being diagnosed due to increased awareness and improved diagnostic techniques, as well as the availability of glucagon-like-peptide-2 (GLP-2) analogs indicated for the treatment of SBS [39], and associated market development efforts. The greatest increase in prevalence over the study period was seen in the pediatric population, corresponding with the approval of a GLP-2 analog for pediatric use [39]. Furthermore, the US population is aging, and older adults are at greater risk of developing SBS due to a higher incidence of medical conditions requiring surgical interventions and poor blood flow or clot formation in the mesenteric vasculature.

In a recent analysis, US healthcare providers revealed a nonuniform management practice of patients with SBS-IF and various knowledge gaps while treating these patients [40]. Additionally, there is a lack of knowledge of chronic IF among US gastroenterologists [41]. Brandt et al. demonstrated that filling the knowledge gap associated with SBS among physicians optimized the use of PN and other disease management tools [7], [21].

The current analysis is a step forward in informing the latest patient demographics using the most stringent claims-based patient identification technique, and the resulting insights could be used to drive medical education around how the physician community might employ criteria to identify patients. Educating healthcare providers on the critical elements of SBS is key to consistently and accurately diagnosing this high-burden disease in the future.

Several limitations of these analyses should be considered. The Komodo Healthcare Map™ claims database does not capture any symptoms, diagnoses, or treatments not reported on a medical claim and submitted for reimbursement. Due to the requirement to have continuously enrolled patients with medical and pharmacy coverage in the calendar year and previous three years, the prevalence estimates were only reported for patients ≥ 3 years of age. Additionally, a lack of standardized coding practices in healthcare settings may have led to inaccurate or incomplete characterization of patient medical conditions. The criteria for identifying patients with SBS-IF utilized information submitted via claims to health insurers, such as ICD-10 codes, potentially resulting in an underestimation of prevalence, particularly if diagnosis codes for malabsorption were not systematically utilized on submitted insurance claims. This is inevitably an under-estimate, although a robust dataset based on stringent criteria. Finally, because of the nature of this claims database analysis, we cannot provide data on uninsured patients with SBS, with means our results only provide an estimate of the prevalence of SBS-IF in the USA.

Despite these limitations, this study provides important information on the most recent prevalence rates of SBS-IF in the USA from 2019 to 2021.

## Conclusion

Using an algorithm that employed stringent identification criteria to accurately identify patients with SB-IF, the annual US prevalence of SBS-IF was estimated at approximately 12,000 patients in 2021, yielding approximately 36 cases per million US inhabitants. Modest increases in prevalence were observed from 2019 to 2021, and further research should be conducted to investigate whether this trend may continue and identify factors that may be driving this increase. This study establishes a robust and reproducible identification algorithm for patients with SBS-IF that can support future insurance claims-based analyses, including evaluation of healthcare resource utilization and associated clinical burden of disease.

## Patient’s/ Guardian’s consent

Not applicable as this is a claims database analysis.

## Ethical clearance

Not applicable as this is a database analysis not directly involving patients.

## Author contributions

GM, JH, and KI contributed to the conception and design of the research and KI contributed to data interpretation. AA, GM, and MG contributed to the analysis and interpretation of the data. All authors agree to be fully accountable for ensuring the integrity and accuracy of the work and have read and approved the final manuscript.

## CRediT authorship contribution statement

**Gail Mitchell:** Formal analysis, Conceptualization. **Mark Gallivan:** Formal analysis. **Jeff Henderson:** Formal analysis, Conceptualization. **Kishore Iyer:** Conceptualization. **Ahan Ali:** Formal analysis.

## Funding

This work was supported by Ironwood Pharmaceuticals, Inc., Boston, MA, USA.

## Declaration of Competing Interest

These authors disclosure the following:

Ahan Ali reports receiving consultant/advisor fees from Trinity Life Sciences.

Gail Mitchell is an employee of Ironwood Pharmaceuticals, Inc.

Mark Gallivan reports receiving consultant/advisor fees from Trinity Life Sciences.

Jeff Henderson is an employee of Ironwood Pharmaceuticals, Inc.

Kishore R. Iyer reports advisor fees and grant support from North Sea Therapeutics and VectivBio (now part of Ironwood Pharmaceuticals, Inc.); consultant/advisor fees, speaker fees and grant support from Takeda.

## Data Availability

Data described in the manuscript will not be made available because all data available for this analysis are included in the manuscript.
