# Supplementary material for: Epidemiology of US patients with short bowel syndrome-associated intestinal failure: A claims database analysis
Source: Intest Fail. 2025 Nov 10;8:100325. doi: 10.1016/j.intf.2025.100325 (PMC12851070; doi:10.1016/j.intf.2025.100325)
Supplement: Supplementary file 1 — Supplementary material [file mmc1.docx]

Supplementary Appendix

Table of Contents

[Figure S1. Top prescribed medications among patients with SBS-IF in 2021 (% of patients; n=1381) 2](#_Toc175059159)

[Table S1. Most common comorbidities using the Clinical Classifications Software Refined groupings among patients with SBS-IF by age and gender in 2021 – Primary cohort and alternative definitions 3](#_Toc175059160)

[Table S2. Most commonly prescribed medications among patients with SBS-IF by age and gender in 2021 – Primary cohort and alternative definitions 5](#_Toc175059161)

[Table S3. Patient demographics and characteristics in 2021 – Primary cohort and alternative definitions 7](#_Toc175059162)

Figure S1. Top prescribed medications among patients with SBS-IF in 2021 (% of patients; *n*=1381). IF, intestinal failure; SBS, short bowel syndrome.


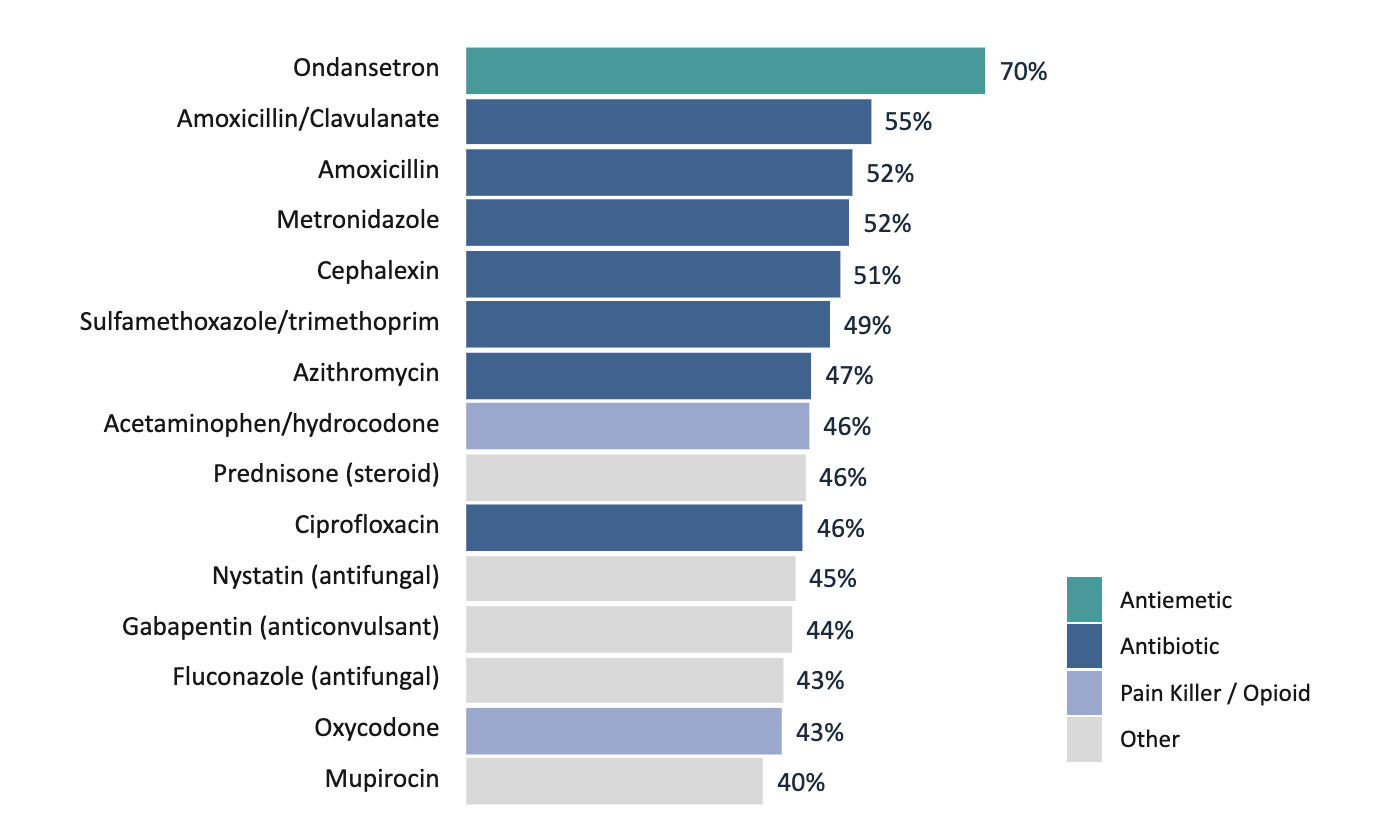


# Table S1. Most common comorbidities using the Clinical Classifications Software Refined groupings among patients with SBS-IF by age and gender in 2021 – primary cohort and alternative definitions

|  | **Males aged**  **3-17 y** | **Males aged**  **18-44 y** | **Males aged**  **45-64 y** | **Males aged**  **65-84 y** | **Males aged**  **≥85 y** | **Females aged**  **3-17 y** | **Females aged**  **18-44 y** | **Females aged**  **45-64 y** | **Females aged**  **65-84 y** | **Females aged**  **≥85 y** |
| --- | --- | --- | --- | --- | --- | --- | --- | --- | --- | --- |
| Primary cohort, *n* (%) | ***n*=223** | ***n*=96** | ***n*=108** | ***n*=41** | ***n*=2** | ***n*=196** | ***n*=298** | ***n*=300** | ***n*=112** | ***n*=5** |
| Gastrointestinal disorders | 196 (88) | 83 (86) | 81 (75) | 28 (68) | 2 (100) | 172 (88) | 253 (85) | 245 (82) | 77 (69) | 4 (80) |
| Abdominal pain | 161 (72) | 72 (75) | 85 (79) | 31 (76) | 2 (100) | 154 (79) | 259 (87) | 257 (86) | 85 (76) | 2 (40) |
| Fluid and electrolyte disorders | 135 (61) | 72 (75) | 85 (79) | 34 (83) | 2 (100) | 118 (60) | 214 (72) | 241 (80) | 94 (84) | 4 (80) |
| Nausea and vomiting | 114 (51) | 44 (46) | 56 (52) | 17 (41) | 1 (50) | 103 (53) | 223 (75) | 199 (66) | 55 (49) | 2 (40) |
| Esophageal disorders | 127 (57) | 48 (50) | 58 (54) | 25 (61) | 2 (100) | 95 (48) | 186 (62) | 202 (67) | 61 (54) | 2 (40) |
| Alternative definition 1, *n* (%) | ***n*=269** | ***n*=150** | ***n*=179** | ***n*=70** | ***n*=7** | ***n*=239** | ***n*=353** | ***n*=352** | ***n*=147** | ***n*=12** |
| Gastrointestinal disorders | 215 (80) | 119 (79) | 133 (74) | 44 (63) | 6 (86) | 206 (86) | 302 (86) | 276 (78) | 107 (73) | 7 (58) |
| Abdominal pain | 193 (72) | 114 (76) | 153 (85) | 52 (74) | 6 (86) | 184 (77) | 310 (88) | 309 (88) | 122 (83) | 9 (75) |
| Fluid and electrolyte disorders | 152 (57) | 106 (71) | 140 (78) | 54 (77) | 6 (86) | 144 (60) | 266 (75) | 267 (76) | 113 (77) | 9 (75) |
| Nausea and vomiting | 133 (49) | 77 (51) | 97 (54) | 35 (50) | 2 (29) | 125 (52) | 273 (77) | 246 (70) | 83 (56) | 1 (8) |
| Esophageal disorders | 160 (59) | 88 (59) | 99 (55) | 48 (69) | 3 (43) | 128 (54) | 232 (66) | 223 (63) | 84 (57) | 5 (42) |
| Alternative definition 2, *n* (%) | ***n*=411** | ***n*=272** | ***n*=360** | ***n*=168** | ***n*=12** | ***n*=396** | ***n*=772** | ***n*=757** | ***n*=265** | ***n*=30** |
| Gastrointestinal disorders | 318 (77) | 182 (67) | 217 (60) | 86 (51) | 8 (67) | 312 (79) | 562 (73) | 519 (69) | 158 (60) | 15 (50) |
| Abdominal pain | 286 (70) | 207 (76) | 282 (78) | 123 (73) | 10 (83) | 294 (74) | 655 (85) | 632 (83) | 208 (78) | 21 (70) |
| Fluid and electrolyte disorders | 221 (54) | 189 (69) | 271 (75) | 124 (74) | 9 (75) | 227 (57) | 552 (72) | 552 (73) | 208 (78) | 26 (87) |
| Nausea and vomiting | 208 (51) | 150 (55) | 187 (52) | 68 (40) | 5 (42) | 222 (56) | 578 (75) | 499 (66) | 144 (54) | 11 (37) |
| Esophageal disorders | 250 (61) | 153 (56) | 199 (55) | 102 (61) | 8 (67) | 227 (57) | 459 (59) | 449 (59) | 157 (59) | 14 (47) |

Differences in most common comorbidities were not significant by gender in the primary and alternative definition 1 cohort. All other age and gender comparisons across all three cohorts were significant (*P* <0.05).

IF, intestinal failure; SBS, short bowel syndrome.

# Table S2. Most commonly prescribed medications among patients with SBS-IF by age and gender in 2021 – primary cohort and alternative definitions

|  | **Males aged**  **3-17 y** | **Males aged**  **18-44 y** | **Males aged**  **45-64 y** | **Males aged**  **65-84 y** | **Males aged**  **≥85 y** | **Females aged**  **3-17 y** | **Females aged**  **18-44 y** | **Females aged**  **45-64 y** | **Females aged**  **65-84 y** | **Females aged**  **≥85 y** |
| --- | --- | --- | --- | --- | --- | --- | --- | --- | --- | --- |
| Primary cohort, *n* (%) | ***n*=222** | ***n*=96** | ***n*=108** | ***n*=41** | ***n*=2** | ***n*=196** | ***n*=298** | ***n*=299** | ***n*=112** | ***n*=5** |
| Ondansetron | 126 (57) | 61 (64) | 73 (68) | 23 (56) | 2 (100) | 108 (55) | 249 (84) | 243 (81) | 83 (74) | 3 (60) |
| Amoxicillin/clavulanate | 118 (53) | 60 (63) | 56 (52) | 22 (54) | 1 (50) | 86 (44) | 170 (57) | 171 (57) | 55 (49) | 3 (60) |
| Amoxicillin | 139 (63) | 46 (48) | 51 (47) | 18 (44) | 0 (0) | 118 (60) | 162 (54) | 142 (47) | 48 (43) | 3 (60) |
| Metronidazole | 107 (48) | 50 (52) | 59 (55) | 16 (39) | 1 (50) | 105 (54) | 173 (58) | 165 (55) | 43 (38) | 1 (20) |
| Cephalexin | 72 (32) | 42 (44) | 57 (53) | 18 (44) | 1 (50) | 70 (36) | 180 (60) | 175 (59) | 64 (57) | 3 (60) |
| Alternative definition 1, *n* (%) | ***n*=268** | ***n*=150** | ***n*=179** | ***n*=68** | ***n*=7** | ***n*=239** | ***n*=353** | ***n*=352** | ***n*=146** | ***n*=11** |
| Ondansetron | 139 (52) | 96 (64) | 125 (70) | 38 (56) | 3 (43) | 143 (60) | 290 (82) | 289 (82) | 107 (73) | 4 (36) |
| Amoxicillin/clavulanate | 143 (53) | 91 (61) | 89 (50) | 34 (50) | 4 (57) | 117 (49) | 220 (62) | 210 (60) | 70 (48) | 4 (36) |
| Amoxicillin | 164 (61) | 80 (53) | 74 (41) | 24 (35) | 1 (14) | 145 (61) | 196 (56) | 165 (47) | 65 (45) | 5 (45) |
| Metronidazole | 119 (44) | 72 (48) | 89 (50) | 27 (40) | 2 (29) | 100 (42) | 180 (51) | 202 (57) | 50 (34) | 2 (18) |
| Cephalexin | 94 (35) | 66 (44) | 87 (49) | 32 47) | 3 (43) | 99 (41) | 212 (60) | 195 (55) | 82 (56) | 3 (27) |
| Alternative definition 2, *n* (%) | ***n*=410** | ***n*=271** | ***n*=360** | ***n*=166** | ***n*=12** | ***n*=396** | ***n*=772** | ***n*=756** | ***n*=262** | ***n*=28** |
| Ondansetron | 226 (55) | 185 (68) | 268 (74) | 107 (64) | 6 (50) | 248 (63) | 669 (87) | 644 (85) | 198 (76) | 13 (46) |
| Amoxicillin/clavulanate | 239 (58) | 166 (61) | 192 (53) | 82 (49) | 6 (50) | 226 (57) | 466 (60) | 440 (58) | 130 (50) | 12 (43) |
| Amoxicillin | 263 (64) | 141 (52) | 183 (51) | 74 (45) | 3 (25) | 261 (66) | 451 (58) | 380 (50) | 119 (45) | 10 (36) |
| Metronidazole | 142 (35) | 117 (43) | 153 (43) | 55 (33) | 2 (17) | 129 (33) | 393 (51) | 420 (56) | 96 (37) | 5 (18) |
| Cephalexin | 159 (39) | 122 (45) | 188 (52) | 82 (49) | 5 (42) | 183 (46) | 488 (63) | 419 (55) | 138 (53) | 13 (46) |

Differences in most commonly prescribed medications were significant by gender and age group in all cohorts.

IF, intestinal failure; SBS, short bowel syndrome.

# Table S3. Patient demographics and characteristics in 2021 – primary cohort and alternative definitions

|  | **Primary cohort**  **(*n*=1381)** | **Alternative definition 1**  **(*n*=1778)** | **Alternative definition 2**  **(*n*=3443)** |
| --- | --- | --- | --- |
| Age (*n*, %) |  |  |  |
| 3-17 years | 419 (30) | 508 (29) | 807 (23) |
| 18-44 years | 394 (29) | 503 (28) | 1044 (30) |
| 45-64 years | 408 (30) | 531 (30) | 1117 (32) |
| 65-84 years | 153 (11) | 217 (12) | 433 (13) |
| 85+ years | 7 (1) | 19 (1) | 42 (1) |
| Gender (*n*, %) |  |  |  |
| Female | 911 (66) | 1103 (62) | 2220 (64) |
| Male | 470 (34) | 675 (38) | 1223 (36) |
| Race (*n*, %) |  |  |  |
| White | 669 (48) | 786 (44) | 1578 (46) |
| Black or African American | 140 (10) | 212 (12) | 428 (12) |
| Asian/Pacific Islander | 20 (1) | 29 (2) | 52 (2) |
| American Indian/Alaskan Native | 1 (0) | 3 (0) | 5 (0) |
| Other | 43 (3) | 64 (4) | 102 (3) |
| Unknown | 508 (37) | 684 (38) | 1278 (37) |
| Ethnicity (*n*, %) |  |  |  |
| Hispanic or Latino | 99 (7) | 251 (7) | 251 (7) |
| Not Hispanic or Latino | 586 (42) | 1494 (43) | 1494 (43) |
| Unknown | 696 (50) | 1698 (49) | 1698 (49) |
| Comorbidities (*n*, %)^a^ |  |  |  |
| Gastrointestinal disorders | 1141 (83) | 1415 (76) | 2377 (69) |
| Abdominal pain | 1108 (80) | 1452 (82) | 2718 (79) |
| Fluid and electrolyte disorders | 999 (72) | 1257 (71) | 2379 (69) |
| Nausea and vomiting | 814 (59) | 1072 (60) | 2072 (60) |
| Esophageal disorders | 806 (58) | 1070 (60) | 2018 (59) |
| Top prescribed medications (*n*, %)^b^ |  |  |  |
| Ondansetron | 971 (70) | 1234 (69) | 2564 (74) |
| Amoxicillin/clavulanate | 742 (54) | 982 (55) | 1959 (57) |
| Amoxicillin | 727 (53) | 919 (52) | 1885 (55) |
| Metronidazole | 720 (52) | 843 (47) | 1512 (44) |
| Cephalexin | 682 (49) | 873 (49) | 1797 (52) |

^a^Occurred in ≥60% of patients in each cohort.

^b^Prescribed in >50% of patients in each cohort.

Differences in patient demographics and characteristics were significant by age *(P* <0.0001), comorbidities (*P* <0.01) and most prescribed medications (*P* <0.01).
